# Assessment of selected nutrients and toxic metals in fruits, soils and irrigation waters of Awara Melka and Nura Era farms, Ethiopia

**DOI:** 10.1186/s40064-016-2382-3

**Published:** 2016-06-16

**Authors:** Shambel G. Yami, Bhagwan Singh Chandravanshi, Taddese Wondimu, Cherinet Abuye

**Affiliations:** Department of Chemistry, College of Natural Sciences, Addis Ababa University, P. O. Box 1176, Addis Ababa, Ethiopia; Department of Chemistry, Johannesburg University, P. O. Box 524, Auckland Park, Johannesburg, 2006 South Africa; Nutritional and Food Science Department, Ethiopian Health and Nutritional Research Institute, P. O. Box 1242, Addis Ababa, Ethiopia

**Keywords:** Banana, Grape, Guava, Mandarin, Orange, Fruits, Minerals, Toxic metals, Ascorbic acid, Farm soils, Irrigation waters

## Abstract

The objective of the present study was to determine the levels of essential nutrients (Na, K, Ca, Mg, Fe, Cu, Zn, Mn) and toxic metals (Pb and Cd) in selected varieties of fruits (banana, grape, guava, mandarin and orange), farmland soils and irrigation waters to assess nutritional impact on health. The freeze dried fruits were digested using HNO_3_ and HClO_4_ and the levels of the elements determined using flame atomic absorption spectrometer. The highest amount of K (3480) and Mg (281) and the lowest amount of Na (6.4) and Ca (75) µg/g were obtained in banana compared to other analyzed fruits. Cd was detected in guava (0.2 µg/g), orange (0.3 µg/g) and mandarin (0.1 µg/g), but not detected in banana and grape. Pb was not detected in all the fruits. Appreciable amount of total ascorbic acid was found in guava (1170 µg/g) compared to that of other fruits. Furthermore, farmland soils and irrigation waters were analyzed for the selected metals and physico-chemical properties of soils including pH, electrical conductivity, cation exchange capacity, texture and available phosphorus determined to elucidate factors controlling the availability of metals in fruits. Applying statistical packages, analysis of variances and Pearson correlation, links between metal levels in fruits and those in soil and water samples were evaluated.

## Background

Fruits are natural staple food for humans. They contain substantial quantities of essential nutrients in good proportion (Elbagermi et al. 2012). Other vital components in fresh fruits such as vitamin C, carotenoids, minerals and dietary fiber are vital requisites to body’s optimum immunity functions (Maggini et al. 2010). In addition, they contain water, calcium, iron, for the maintenance of health and the prevention of various diseases (D’Mello 2003). Consumption of fruits on a regular basis is critical in providing health promoting nutrients to the human body.

Studies reporting the mineral contents of fruits and vegetables have appeared in the literature in an attempt to assess the nutritional benefits and potential risks arising from the consumption of fruits. Kalagbor et al. (2014) have studied the levels of heavy metals in four fruits from Nigeria. Basha et al. (2014) have reported trace metals in vegetables and fruits cultivated in India. Igwegbe et al. (2013) have reported a survey of heavy metal contents of selected fruit and vegetable crops in Nigeria. Elbagermi et al. (2012) have reported heavy metal content in fruits and vegetables in Libya. Akhtar et al. (2010) have studied heavy metal content of mangoes cultivated in different regions of Pakistan. Ang and Ng (2000) have reported trace element concentration in mango, seedless guava and papaya grown in Malaysia. Mausi et al. (2014) have conducted study on the assessment of selected heavy metal concentrations in selected fresh fruits in Kenya.

Mineral and nutritional analysis has been reported on apple, avocado, apricot, and banana collected from Tigray, Gonder, Wello, Arsi, Harar and Sidamo (Ethiopia) and average data for each fruit have been reported (EHNRI 1998). However, variations of nutrient composition of fruits on characteristics of the land, climate, cultivation conditions, and stage of maturation, and the role of fertilization and irrigation water have been overlooked. In addition, only few fruits were analyzed even though the composition varies within species.

In recent years, growing interest has been shown in the levels of metals in vegetables and other food stuffs, farmland soils and irrigation waters in Ethiopia. Yemane et al. (2008) studied the levels of essential and non-essential metals in tea leaves and soils of Wushwush farms. Nigussie et al. (2010) established correlation among trace metals in fish, sediment and water samples of lakes Awassa and Ziway. Atlabachew et al. (2011) profiled major, minor and toxic metals in soil and khat cultivars. Weldegebriel et al. (2012) determined the levels of metals in vegetables grown in soils irrigated with river water in Addis Ababa. The study by Kitata and Chandravanshi (2012) reports the levels of major and trace metals in onion and irrigation water around Meki Town and Lake Ziway. More recently, the levels of essential and non-essential metals in ginger (Wagesho and Chandravanshi 2015) and in seeds of cardamom, also locally known as korerima, cultivated in Ethiopia were reported (Mekassa and Chandravanshi 2015).

There are variations in nutrient composition among and within the existing species of fruits depending on the characteristics of the land, climate, cultivation conditions and composition of irrigation water. The United Nations Organization for Food and Agriculture has recommended that food composition charts should be prepared for food produced and consumed locally. In Ethiopia, such data are not readily available. Therefore, the objective of this study was to determine the extent of accumulation of essential nutrients (Na, K, Ca, Mg, Fe, Cu, Zn, and Mn), toxic metals (Pb and Cd), moisture content and total ascorbic acid in fruits (banana, grape, guava, mandarin and orange) produced in Awara Melka and Nura Era Farms, Ethiopia. The soils and irrigation waters were also analyzed for metals to estimate relationship between the levels of metals in fruits with that of soil and irrigation water.

## Methods

### Equipment

Freeze dryer (Freeze dry-3, Labconco, Kentucky, USA) was used for drying fruit samples to constant mass. A blender (Super Blender Mill Grater 3, France) was used to homogenize fruit samples. A digestion apparatus consisting of 100 mL round or flat bottom flask fitted with condenser and a hotplate was used for decomposing the sample matrix. A flame atomic absorption spectrometer (Buck Scientific Model 210VGP AAS, East Norwalk, USA) was used for determining the concentrations of metals in fruit, soil and water samples. S2000 Lightwave Spectrophotometer (Cambrige, UK) equipped with UV/VIS diode array detector was used for the determination of total ascorbic acid. Inolab pH/ION Level 2 pH meter (WTW, Weilheim, Germany) was used for the measurement of the soil pH. Thermo Orion Conductivity meter Model 145 (Thermo Electron Corporation, Midrand, USA) was used for the measurement of electrical conductivity of soil samples.

### Reagents

Deionized water was used throughout the experiments for all dilutions and rinsing purposes. 70 % HNO_3_ (Spectrosol^®^, England), 70 % HClO_4_ (Aldrich, A.C.S. Reagent, Germany) and 37 % HCl (Riedel–deHaen, Chem Pure, Germany) were used as received for the digestion of fruit and soil samples. Stock standard solutions of the metals (1000 mg/L) calibration standards (Buck Scientific, USA), prepared as nitrates for each element in 2 % HNO_3_, were used for the preparation of calibration curves for the determination of metals in the samples. 98.5 % trichloroacetic acid (Hopkin and Williams, A.C.S. Reagent, England), meta-phosphoric acid (BDH, G.R., England), 2,4-DNPH (Schiapparelli, A.R., Italy), 95–97 % H_2_SO_4_ (Merck, Germany), 99.5 % thiourea (Merck, G.R., Germany), 99.5 % Br_2_ (Merck, A.R., Germany) and 99.7 % l-ascorbic acid standard (BDH, AnalaR^®^, England) were used for the determination of vitamin C in the fruit samples.

### Fruits sampling and pretreatment

**Fig. 1:**
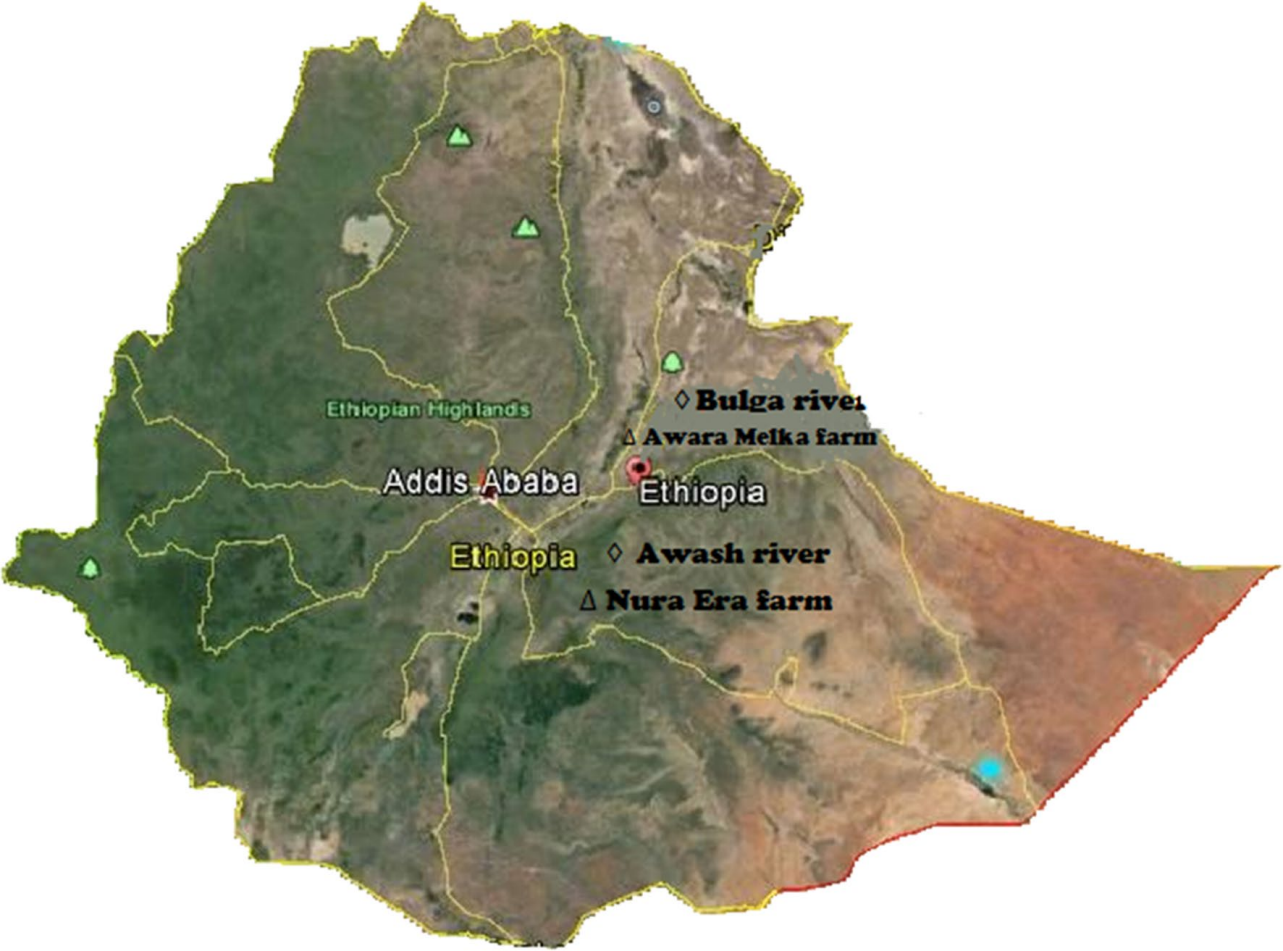
Map of Ethiopia showing the sampling sites

The selection of fruit type among the available varieties was based on the largest productivity and high consumption of the fruit by the society. The largest citrus orchard (about 1000 ha) in Ethiopia is found in Nura Era Farm (Seifu 2003). Banana is not produced in Nura Era farm. Hence unripe banana (*Musa cavendish*) was collected from Awara Melka Farm and allowed to ripen in the laboratory according to the procedure followed by the farm (providing heat by covering the fruit with sacks and plastics). White grape, guava (unknown variety), mandarin (orlando variety), and orange (pineapple variety) of similar degree of maturity were collected randomly from different plants grown in Nura Era Farm. All the fruits were fully matured and first grade according to the farms’ criteria. The sampling sites are shown in Fig. 1.

The fruits except banana were placed in ice-box to keep them as fresh as possible with polyethylene sheet between each fruits to minimize diffusion through contact. The fruits were transported to the laboratory within 1 day after collection. Surface contaminants of the fruits were washed with tap water, rinsed with deionized water and dried with tissue paper (Storey and Treeby 2000). Some portions of the fruits were kept at −20 °C until analysis for ascorbic acid without peeling the fruits. From the remaining fruits, each fruit was dissected into quarters along the equatorial plane (Townshend 1995) with plastic knife. A quarter or a representative sub-sample of each fruit of the same species was collected and peeled. Seeds and large particles of cellular materials were removed to represent the edible portion (Miller-Ihli 1996; Simpkins et al. 2000). The samples were homogenized in a blender and freeze dried until constant masses were obtained. The dry samples were stored in polyethylene bags until the time of analysis.

### Soil sampling and pretreatment

Two soil samples from Awara Melka Farm (one from banana land and one from orange and mandarin land) and two soil samples from Nura Era Farm (one from grape land and one from guava land) were collected. Sampling points for soil samples were selected based on an imaginary zigzag line/pattern (Tan 1996). About 500 g of soil from each of the four composite samples was taken and thoroughly mixed in the laboratory. The soil sample was air dried in the laboratory, ground using mortar and pestle, sieved through 0.090 mm sieve and then stored in plastic bags until analysis.

### Water sampling and pretreatment

Water samples were collected from Bulga and Awash Rivers that are used for irrigation by Awara Melka and Nura Era Farms, respectively. These two rivers are approximately 1 km from the respective farms. The samples were collected at inversion points of the main channel from the rivers and from the channels (four samples from Awara Melka Farm and nine from Nura Era Farm at the inversion from sub-main irrigation channel to each block of fruit lands) that feed the selected fruits. In all cases the water samples were taken by placing the mouth of polyethylene containers (nitric acid cleaned) approximately 2 cm below the surface of the water. The water samples were brought to the laboratory and then centrifuged to remove the suspended particles, filtered, and preserved with 2 mL of 10 % HNO_3_ per 100 mL of water and stored in a refrigerator (Fifield and Haines 1997).

### Digestion of fruits for metal determination

The optimal procedure was developed with banana and orange samples, expecting that the citrus fruits have similarity with that of orange. Different wet-digestion methods were tested to select optimum procedure. The procedure was adopted from that used by Clemson University with some modification (Clemson University 2000). The optimization was based on production of clear solution, shorter digestion time and minimum reagent consumption. The optimum procedure was found to be the one involving refluxing 0.25 g of fruit sample in a 100 mL round bottomed flak with 4 mL concentrated HNO_3_ and 4 mL of concentrated HClO_4_ for 125 min at 240 °C. The cooled digest and its washings were transferred into a 25 mL volumetric flask and diluted to the mark with deionized water. The digests were prepared in triplicates for each sample.

### Extraction of metal from soil samples

Conventional aqua regia digestion was applied (Chen and Ma 2001) using 100 mL glass Erlenmeyer flask. Well-mixed soil sample weighing 0.5000 g was digested with 12 mL of aqua regia on a boiling water bath for 3 h. The mixture was cooled, diluted with 20 mL of 2 % (v/v) nitric acid and transferred into a 100 mL volumetric flask after filtering through Whatman No. 1 filter paper and diluted to 100 mL with deionized water.

### Determination of metals in fruits, soils and water samples

The concentrations of Ca, Mg, Fe, Mn, Cu, Zn, Pb and Cd in the digested fruit and soil samples, and pretreated water samples were determined using flame atomic absorption spectrometer. An appropriate dilution was made with 0.5 % (w/w) Sr(NO_3_)_2_ to overcome ionic interference during the determination of Ca and Mg (Paul and Shaha 2004). However the concentrations of Na and K were determined in emission mode of FAAS with optimum instrumental condition (Paul and Shaha 2004). Metal concentrations were determined in the digested solutions using external calibration curves.

### Recovery of metals in spiked fruit samples

The validity of optimized digestion procedure for fruits was checked by carrying out spiking test and evaluating percent recovery. This involved spiking of fruit samples with standard solutions of metals (Maroto et al. 2001). To do this, a fixed volume of a mixture of metal standards was added to a mixture of 0.25 g of fruit and then the optimized procedure for fruit digestion was carried out on this mixture. The digests were prepared in triplicates. The concentrations of the metals were determined with FAAS using external calibration graph. The percentage recoveries were found within acceptable range (100 ± 10) for all the elements, except Mg, for which 89 % recovery was obtained. These findings demonstrated suitability of the digestion procedure developed.

### Recovery of metals in spiked soil samples

To check the validity of the optimized digestion procedure for the determination of metals in soil samples, spiking tests were conducted. To do this, a fixed volume of a mixture of metal standards was added to a mixture of 0.5000 g of soil and 12 mL aqua regia. The selected digestion procedure was carried out on this mixture as it was done for the unspiked sample. The digests were prepared in triplicates. The concentrations of the metals were determined with FAAS using external calibration graph. The percentage recoveries were found within acceptable range (100 ± 10) for all the elements, except for K (77.5 %) and Na (75 %). Indeed, low recoveries are expected for Na and K, because these metals cannot be fully extracted from silicate lattice (Chen and Ma 2001) using aqua regia.

### Extraction and determination of vitamin C in fruit samples

Total ascorbic acid (vitamin C) content was determined by 2,4-dinitophenylhydrazine (DNPH) method (Kapur et al. 2012). A homogenized sample (5.0000 g) of the fruit was used for this analysis. To study the recovery of total ascorbic acid using the selected method 0.4 mL of of l-ascorbic acid (1 mg/mL) standard was added to 5.000 g of wet fruit sample to give 0.8 μg ascorbic acid/mL in the final stage of the analysis. The mixture was extracted following the selected analytical procedure. Absorbance of the spiked samples was monitored by a UV/Vis spectrophotometer at a wavelength of 521 nm. Triplicates of analysis were done. A percentage recovery of 92.4 ± 0.2 was found. This confirms that the method is valid for the determination of total ascorbic acid in the fruits.

### Measurement of physico-chemical characteristics of soils

The soil pH, electrical conductivity, texture, organic matter, available phosphorus, and cation exchange capacity (CEC) were determined by the reported methods (Tan 1996; Sertse and Taye 2000).

### Statistical analysis

The analysis of variance for the equality of means and correlation between the elements in the fruit, water and soil samples were done using the SPSS 20.0.

## Results and discussion

### Moisture content of fruits

Results from food analysis are usually expressed on a wet weight basis. Thus it is important that no loss of moisture occur until the sample is weighed. Subsequently the sample can be dried either conventionally or by freeze-drying. Freeze drying is favored by many workers (Van Loon, 1985). Freeze drying ensures high flavor retention and minimal damage to product structure and nutritional value; permits fast and nearly complete dehydration (Francis 2000).

The moisture contents (Table 1) of the fruits were determined by measuring the mass loss of the fruits using freeze-drying unit until constant mass was obtained. As can be seen from Table 1, the moisture content is the lowest for banana and the highest for mandarin. The order of moisture content in the fruits is mandarin > orange > grape > guava > banana. These values are comparable with literature values (Sanchez-Castillo et al. 1998; Francis 2000; Li and Anderw 2002).

**Table 1:** The moisture content of fruits (mean ± SD, n = 9)

Fruit	Moisture (%)			
	Found in this study (Ethiopia)	Literature value	Country	Reference
Banana	74.9 ± 0.2	76.0	Netherlands	Francis (2000)
Grape	82.5 ± 0.2	83.0	Netherlands	Francis (2000)
		85.3	Bangladesh	Paul and Shaha (2004)
		81.5	Mexico	Sanchez-Castillo et al. (1998)
Guava	79.7 ± 0.4	81.0	Netherlands	Francis (2000)
Mandarin	88.4 ± 0.4	88.0	Netherlands	Francis (2000)
Orange	85.9 ± 0.3	87.0	Netherlands	Francis (2000)
		87.8	Bangladesh	Paul and Shaha (2004)
		85.5	Mexico	Sanchez-Castillo et al. (1998)

### Concentration of metals in fruits

The metal contents of the fruits are summarized in Table 2.

**Table 2:** Metal levels in fruit samples (mean ± SD, n = 6)

Fruit	Concentration of metals (µg/g edible portion)									
	Na	K	Ca	Mg	Fe	Cu	Zn	Mn	Pb	Cd
Banana	6.4 ± 0.2	3480 ± 20	75.0 ± 1	281 ± 1	4.6 ± 0.1	0.8 ± 0.1	2.3 ± 0.1	1.2±0.1	ND	ND
Grape	13.7 ± 0.3	1780 ± 30	250 ± 1	60.0 ± 1	3.8 ± 0.2	2.6 ± 0.1	2.2 ± 0.1	3.4 ± 0.2	ND	ND
Guava	15.6 ± 0.2	2080 ± 50	481 ± 3	140 ± 2	5.6 ± 0.1	1.2 ± 0.1	1.4 ± 0.1	4.8 ± 0.1	ND	0.200 ± 0.004
Orange	14.7 ± 0.1	1220 ± 20	554 ± 1	79.0 ± 1	2.6 ± 0.1	0.7 ± 0.1	1.6 ± 0.1	1.3 ± 0.1	ND	0.300 ± 0.01
Mandarin	10.6 ± 0.2	1380 ± 20	286 ± 11	84.0 ± 3	2.5 ± 0.1	0.3 ± 0.01	1.2 ± 0.1	0.3 ± 0.01	ND	0.13 ± 0.01

*ND* not detected

The pattern of concentrations of metals in banana is decreased as follows: K > Mg > Ca > Na > Fe > Zn > Mn > Cu. The concentrations of Pb and Cd were below the detection limit of the method. Table 2 indicates that banana can be good source of both major and trace elements that are essential for our body. Especially, banana is a good source of Mg (270 µg/g wet weight) and K (3620 µg/g wet weight) compared to other selected fruits. The obtained concentrations are within the ranges of literature values. For instance the trace element intervals for banana reported in different literatures from 1968 to 1998 for Ca, Mg, Fe, Cu, Zn, Mn, Pb, and Cd (µg/g edible portion) are: 20.0–500, 190–440, 3.4–9.0, 0.7–4.0, 1.5–3.4, and 0.6–14.0, respectively (Hardisson et al. 2001). In another literature, the values that were reported for banana (Cavendish variety) grown in Australia were 3300, 50, 330, 3 and 2 µg/g edible portion for K, Ca, Mg, Fe, and Zn, respectively (Cunningham et al. 2001). These literature values are similar to the results obtained in this study.

The trend in concentrations of metals in grape is K > Ca > Mg > Na > Fe > Mn > Cu > Zn. The average metal concentrations of grape are 13.7 (Na), 1780 (K), 250 (Ca), 60 (Mg), 3.8 (Fe), 2.6 (Cu), 2.2 (Zn) and 3.4 (Mn) µg/g edible portion (Table 2). The obtained results are comparable with literature value (Miller-Ihli 1996). Table 2 shows that copper content of grape is highest compared to other analyzed fruits.

The concentration pattern of the metals in guava is K > Ca > Mg > Na > Fe > Mn > Zn > Cu > Cd. The average concentrations of metals in guava are 15.6 (Na), 2080 (K), 481 (Ca), 140 (Mg), 5.6 (Fe), 1.2 (Cu), 1.4 (Zn), 4.8 (Mn) and 0.2 (Cd) µg/g edible portion. EHNRI (1998) has reported 72 (Na), 2290 (K), 170 (Ca), 6.4 (Fe), 8.4 (Cu) and 2.4 (Zn) µg/g edible portion for guava from Sidamo, Ethiopia and 60 (Na), 2110 (K), 320 (Ca), 30 (Fe), 3.7 (Cu), and 1.4 (Zn) µg/g edible portion for guava from Konso, Ethiopia. These values are nearly similar to those found in the guava from Nura Era Farm except that amount of Ca is higher and amount of Na is lower compared to guava from Konso and Sidamo. In this study it was found that guava contains highest amount of Mn, Na and Fe compared other selected fruits.

The elemental concentration pattern is K > Ca > Mg > Na > Fe > Zn > Mn > Cu > Cd. Ca content of mandarin is about half of the amount in orange. The average concentrations of metals in mandarin are 1380 (K), 286 (Ca), 84 (Mg), 10.6 (Na), 2.5 (Fe), 1.2 (Zn), 0.3 (Cu), 0.3 (Mn) and 0.13 (Cd) µg/g edible portion (Table 2).

The concentration pattern of the metals in orange is K > Ca > Mg > Na > Fe > Zn > Mn ≈ Cu > Cd. There is a similarity in trends of the concentrations of metals in mandarin and orange. The average concentrations of metals in orange are 1220 (K), 554 (Ca), 79 (Mg), 14.7 (Na), 2.6 (Fe), 1.6 (Zn), 1.3 (Mn), 0.7 (Cu) and 0.3 (Cd) µg/g edible portion (Table 2). EHNRI (1998) reported 500 µg/g for Ca and 8 µg/g for Fe in the edible portion of the fruit which is nearly similar to the values obtained in this study. The obtained results are also comparable with literature values other in other reports (Miller-Ihli 1996). Compared to other analyzed fruits, orange contains highest amount of Ca (Table 2).

Generally, for most metals the values are comparable with that reported in literature (Miller-Ihli 1996; EHNRI 1998; Hardisson et al. 2001). Pb was below the detection limit of the method in all the fruits analyzed. This may be because of the fact that Pb forms relatively insoluble minerals in soils (GVRD 1999). The pH of the soils (alkaline) of Awara Melka and Nura Era Farms (Table 3) also favors the insolubility, and therefore unavailability of lead. However, unlike Pb, Cd was detected in guava, orange and mandarin, but its amount was below the detection limit of the method in banana and grape. It has been reported that Cd, Cu and Zn were the main elements that plant could accumulate and pass up to the food chain. The detection of cadmium in the fruits is maybe because of the fact that cadmium ions are readily transferred from the soil to plants, which absorb the element and accumulate it to different degrees, depending on the species (Cabrera et al. 1992).

**Table 3:** Physicochemical characteristics of the soils collected from the Awara Melka and Nura Era farms

Farm	Lands from which soil sample collected	pH	EC (µ Scm^−1^)	OM (%)	Clay and texture (%)	Available P (mg/kg)	CEC (meq/kg)
Awara Melka	Banana land	8.11	185 ± 2	1.55	40.3 (Clay loam)	6.08	452
Nura Era	Orange, mandarin land	7.87	319 ± 3	2.68	45.2 (Clay)	3.68	459
	Grape land	8.23	174 ± 2	1.52	37.8 (Clay loam)	4.48	367
	Guava land	8.00	184 ± 4	2.84	45.4 (Clay)	4.00	472

Samples were analyzed for the above characteristics at Agricultural Research Center, Debre Zeit, Ethiopia, except for EC and pH. Soil pH (H_2_O) was measured at 25 °C (1:2.5) whereas EC at 25 °C (1:5); n = 2 in both cases

### Some characteristics of soil

The impact of contaminated elemental uptake by plant roots is dependent upon many factors, including: the magnitude and chemical forms of trace element(s) present; soil pH, moisture, aeration, temperature, organic matter and phosphate content; the presence or absence of other competing ions; the plant species; rooting depth, age; and seasonal growth effects (Fifield and Haines 1997). The physicochemical characteristics of the soils measured in this study are given in Table 3.

The pH values of the soil samples range from 7.87 to 8.23 which shows the soils of Awara Melka and Nura Era Farms are slightly alkaline (Table 3). Therefore, the availability of the trace elements is low in soils of Awara Melka and Nura Era Farms if only pH is considered. But availability of elements depends on other factors, therefore it is difficult to generalize that the availability of the elements in the soil are high. However one can say that the pH of the soil may contribute for the decrease in availability of the elements. Comparatively, the pH of orange and mandarin land is low compared to other lands (Table 3).

Table 3 shows that the organic contents of soil samples are low (1.52–2.84 %). These values show that the soils of Awara Melka and Nura Era Farms are mineral soil (University of Florida 2005). Information of organic content of soil does not provide much quantitative information to the farmer that is helpful in managing soils for crop production. Instead, the information is generally evaluated on a relative or comparative basis. For instance soils with a higher organic matter will have a higher cation exchange capacity and higher water holding capacity than soil with a lower organic matter (Allan Bande et al. 2002).

It was found that the electrical conductivity of soil from Awara Melka and Nura Era Farms ranges from 174 to 319 µScm^−1^, which shows the EC of the soils are low. The results, therefore, show that amount of ions in the soil are less. The EC of banana, grape, and guava lands are 185, 174, 184 µScm^−1^ while that of orange and mandarin lands is 319 µScm^−1^, which indicates there is much dissolved metals in the orange and mandarin land.

The CEC of the soil samples ranged from 367 to 472 meq/kg dry soil (Table 3). The CEC of grape land is lowest compared to other lands. CEC of soil from guava land is high (472 meq/kg dry soil) compared to soil samples from other lands. This may be the cause for higher accumulation of Na, Fe and Mn in guava (Table 2).

High phosphorus concentrations in the soil decrease Zn availability, as do elevated soil concentrations of Fe, Cu and Ca (Cabrera et al. 1992). Phosphorus is strongly bound to particular soil minerals including aluminum and iron oxides. Table 3 shows that the available phosphorus in Awara Melka soil is higher compared to that of Nura Era soil.

Generally, the soils collected from Awara Melka and Nura Era Farms are similar in most of the soil characteristics (Table 3).

### Concentrations of metals in the soils

Amount of metals in the soils collected from each lands in which fruits were planted are given in Table 4. The concentrations of the selected metals vary from farms to farm and lands to land in which fruits are grown. For this study the soil sample were categorized according to lands/blocks in which the fruits are grown. For instance the soil that was taken from blocks in which banana is grown (named in this case banana land) was analyzed separately. The results are given separately for each land of fruit. Soils from orange and mandarin land were pooled together because they are adjacent to each other and furthermore, application of fertilizers, insecticides, pesticides and irrigation programs are almost the same (Seifu 2003).

**Table 4:** Average metal concentration in soils from Awara Melka and Nura Era Farms, (mean ± SD, n = 6)

Farm	Fruit land	Concentration of metals in soils (mg/kg dry soil)									
		K	Ca	Mg	Fe	Mn	Cu	Zn	Pb	Cd	Na
Awara Melka	Banana land	5020 ± 50	13,200 ± 200	8400 ± 100	61,600 ± 200	2440 ± 60	32.7 ± 0.5	111 ± 1.4	15.3 ± 0.6	3.54 ± 0.16	211 ± 2.8
Nura Era	Orange, mandarin land	7500 ± 170	16,500 ± 200	7700 ± 300	42,800 ± 1500	2950 ± 90	17.3 ± 0.9	130 ± 1.3	31.3 ± 1.7	6.23 ± 0.40	125 ± 5.1
	Grape land	9070 ± 80	7900 ± 100	6400 ± 100	38,800 ± 100	2940 ± 110	25.6 ± 0.9	114 ± 2.3	32.2 ± 0.9	2.54 ± 0.20	51.8 ± 3.3
	Guava land	6680 ± 110	10,400 ± 300	4600 ± 200	26,800 ± 200	2300 ± 80	23.1 ± 1.1	128 ± 1.7	22.9 ± 0.5	3.69 ± 0.13	70.2 ± 3.3

As can be seen from Table 4, sodium is higher in Awara Melka (banana land) soil compared to soils from Nura Era Farms (other lands of fruits). On the other hand, the average concentrations of K in soils from banana, orange and mandarin, grape, and guava lands are 5020, 7500, 9070, and 6680 mg/kg dry soil, respectively. These values are nearly similar to each other. However, average concentration of Ca is different from land to land; highest in orange and mandarin land (16,500 mg/kg dry weight) and lowest in grape land (7900 mg/kg dry weight). The average concentrations of Mg in the soils are 8400, 7700, 6400, and 4600 mg/kg dry weight in banana, orange and mandarin, grape and guava lands, respectively.

Generally, the above metals are very abundant in nature in soils compared to trace elements. Therefore, their variation could be also large from lands to lands. The use of fertilizers could be also a source for these metals.

The average concentration (mg/kg dry soil) of Fe in the soil samples from banana land (Awara Melka), orange and mandarin, grape and guava land are 61,600, 42,300, 38,800 and 26,800, respectively (Table 4). Table 4 shows that there is highest concentration of Fe in Awara Melka Farm (banana land) compared to Nura Era Farm. Even though the values are in the range that has been reported for surface soil, it is above the maximum allowable level (1500 mg/kg) (FAO 1998). The average concentrations of Mn in the soil samples are also above the allowable value (2000 mg/kg) (Itana 2002). The average concentrations of Mn range from 2440 to 2950 mg/kg dry weight (Table 4). These values indicate that there is much amount of Mn in soils of Awara Melka and Nural Era Farms. The average concentration of copper in the sampled soils ranged from 17.3 (orange and mandarin land) to 32.7 (banana land) mg/kg dry soil. The values are in range reported for surface soil (5–80 mg/kg). The concentrations of Cu in the sampled soils are also below maximum allowable level (100 mg/kg) (Itana 2002). The average concentration of Zn in soil samples is almost equal in orange and mandarin land (130 mg/kg dry soil) and guava land (128 mg/kg dry soil). Similarly, average concentrations of Zn in banana and grape land are 111 and 114 mg/kg dry soil, respectively. In all cases the amount of Zn in the soil samples are below the maximum allowable level (300 mg/kg) (Itana 2002).

The average concentrations of Pb in soil samples are found higher in Nura Era Farm compared to that of Awara Melka Farm. The values are in the ranges that are reported for surface soil (Fifield and Haines 1997; Tokaliouglu et al. 2003). All the values are below the maximum allowable level (100 mg/kg) (Tokaliouglu et al. 2003). The average concentration of Cd in soil samples are above is the maximum allowable level (3 mg/kg) (Itana 2002) except for soils from grape land. The most probable source of Cd is phosphates fertilizers and may be pesticides.

Generally the concentrations of Cu, Zn, and Pb in soil samples are in the ranges that have been reported for surface soils. To some extent the concentration of Cd, and to most extent the concentration of Mn and Fe are above the ranges reported for surface soils (Fifield and Haines 1997; Tokaliouglu et al. 2003). The concentration of Cu in soil from Awara Melka is higher compared to that of Nura Era Farm soil samples. The reverse is true for Zn. Concentrations of Pb, is higher at Nura Era Farm compared to that of Awara Melka. Cd is higher in lands in which orange and mandarin are planted. Relatively Zn is higher in soil from Nura Era Farm (Table 4).

### Concentrations of metals in the water samples

The concentrations of metals in the irrigation water collected from the farm channels are generally higher than waters collected from the rivers from which the irrigation water is taken (Table 5). This can be from the soil, fertilizers and pesticides added to the soil.

**Table 5:** Average metal concentration and their ranges in water samples collected from Rivers and Channels used to irrigate Awara Melka and Nura Era Farms (mean ± SD, n = 4)

Farm	River/irrigation channels	Concentration of metals (mg/L)									
		Na	K	Ca	Mg	Fe	Cu	Zn	Mn	Pb	Cd
Awara Melka	Bulga river	20.8 ± 0.2	13.3 ± 0.4	28.0 ± 0.1	11.8 ± 0.1	0.038 ± 0.001	0.024 ± 0.001	0.026 ± 0.001	0.068 ± 0.001	ND	0.0043 ± 0.001
	Channels										
	Mean	28.3 ± 0.3	15.1 ± 0.4	38.4 ± 0.1	12.5 ± 0.6	0.036 ± 0.004	0.046 ± 0.005	0.022 ± 0.002	0.080 ± 0.004	ND	0.0075 ± 0.0003
	Range	26.9–29.0	11.1–24.3	22.2–34.4	8.3–17.4	0.032–0.037	0.043–0.048	**<**0.001–0.037	0.062–0.096	ND	0.0073–0.0078
Nura Era	Awash river	14.9 ± 0.2	13.2 ± 0.6	26.9 ± 0.1	6.7 ± 0.1	0.142 ± 0.012	0.037 ± 0.002	0.092 ± 0.002	0.106 ± 0.002	ND	ND
	Channels										
	Mean	16.3 ± 0.5	15.2 ± 0.6	28.6 ± 0.4	6.8 ± 0.2	0.352 ± 0.011	0.044 ± 0.005	0.061 ± 0.005	0.090 ± 0.006	ND	0.0070 ± 0.004
	Range	12.8–18.8	12.7–19.7	22.0–33.1	5.8–8.0	0.124–0.709	0.011–0.066	0.012–0.184	0.053–0.125	ND	<0.0001–0.0091

*ND* not detected

Awash River contains higher amounts of Fe, Cu, Zn, Mn, and Cd than Bulga River. Similar pattern was obtained for these metals in irrigation water from channels. From agricultural point of view, the maximum concentration limits of metals in irrigation waters are (µg/L): 17 (Cu), 2000 (Zn), 200 (Mn), 65 (Pb) and 10 (Cd) (Itana 2002). The concentrations of Zn, Mn, Pb, and Cd in the waters from the rivers are below the maximum limit. But Cu, in the river water is slightly higher than the maximum recommended limit.

### Total ascorbic acid in the fruits obtained from the selected farms

The concentrations of ascorbic acid in the fruits have been determined from the external calibration curve (R^2^ = 0.9994) obtained with standard solution. From Table 6, the ascorbic acid content ranges from 51 (grape) to 1170 µg ascorbic acid/g edible portion (guava). This indicates that guava is rich in ascorbic acid contents. This has been also shown by many literature reports (Paul and Shaha 2004). Orange is also shown to be a good source of ascorbic acid compared to the other fruits analyzed.

**Table 6:** Average concentrations of total ascorbic acid in the fruits (mean ± SD, n = 6)

No.	Fruit	Concentration (µg AA/g edible portion)			
		Found in this study (Ethiopia)	Literature values	Country	Reference
1	Banana	90 ± 3	127	USA	Wall (2006)
2	Grape	51 ± 1	230	Bangladesh	Paul and Shaha (2004)
3	Guava	1170 ± 60	4000	South Africa	UHIS (2001)
4	Mandarin	193 ± 7	340	Australia	Mitchell et al. (1992)
5	Orange	282 ± 11	620	Bangladesh	Paul and Shaha (2004)

It is pointed out that the tropical guava, *Psidium guajava*, is regarded as an excellent source of vitamin C, but there is greater variation in vitamin C level amongst the various cultivated varieties. For example, the variety “Donaldan” has 3720 µg/g flesh, but variety, ‘Supreme’ has only 440 µg/g edible portion (UHIS 2001). Some South African pink guava cultivars grown for the canning industry are said to have an astonishing 4000 µg/g edible portion (UHIS 2001). The origin of this variation can be due to difference in the variety of the fruits, climate, maturity at which the fruits collected, handling during collection, storage and methods used for the analysis.

### Comparison of metal concentration in soil, water and fruit samples

The concentration of metal in soil (mg/kg), water (mg/L) and fruit (mg/kg) samples are given in Table 7. The accumulation factors of metals from soil to water and from water to fruit samples are calculated by taking the highest concentration (the upper end of the range) of the metals in the soil, water and fruit samples (Table 7). The data in Table 7 clearly show that all the metals are present in the soils at much higher concentrations than in the water samples. This indicates that the metals are mostly present in the soils as insoluble species and hence their accumulation/mobilization factors (from soil to water) are much low (≤0.002) except for Na. In contrast to this, the concentrations of metals in the fruits are much higher than in the water samples. The accumulation factors of metals (from water to fruit) are much higher (8–143) except for Na whose accumulation factor is less than one (0.37). Among the other nine metals, accumulation factors vary from metal to metal, Fe has the lowest accumulation factor (7.9) while K has the highest accumulation factor (143). The differences in the accumulation factors may be due to differences in the ability of individual metals to form complexes with different formation/stability constants.

**Table 7:** Comparison of metal concentrations in the soil, water and fruit samples

Metal	Concentration range in soil (mg/kg)	Concentration range in water (mg/L)	Concentration range in fruit (mg/kg edible part)	Accumulation factor from soil to water	Accumulation factor from water to fruit
Na	51.8–211	12.8–29.0	6.40–10.6	0.137	0.366
K	5020–9070	11.1–24.3	1220–3480	0.00268	143
Ca	7900–16,500	22.0–38.4	75.0–554	0.00233	14.4
Mg	5600–8400	5.80–17.4	60.0–281	0.00207	16.1
Fe	26,800–61,200	0.032–0.709	2.5–5.6	0.0000438	7.90
Cu	17.3–32.7	0.011–0.066	0.3–2.6	0.00202	39.4
Zn	111–128	<0.001–0.184	1.2–2.3	0.00144	12.5
Mn	2300–2950	0.053–0.125	0.3–4.8	0.0000424	38.4
Cd	2.5–6.2	<0.0001–0.0091	<0.1–0.3	0.00147	33.0
Pb	15–32	–	–	–	–

### Analysis of variance

Analysis of variance (ANOVA) is widely used statistical methods to compare the mean of more than two groups of sample. ANOVA uses the F statistic to compare whether the differences between sample means are significant or not (Miller and Miller 2005).

In this study, five fruit samples were collected from two different areas and the metal levels of each sample was analysed by FAAS. During the processes of sample preparation and analysis a number of random errors may be introduced in each aliquot and in each replicate measurement. The variation in sample mean of the analyte was tested by using ANOVA, whether the source for variation was experimental procedure or heterogeneity among the samples.

The ANOVA results (Table 8) showed that there exist statistically significant differences at 95 % confidence level in mean concentrations of all the metals. The source for this significant difference between sample means may be the difference in mineral contents of soil, pH of soil, water, atmosphere; variation in application of agrochemicals like fertilizers, pesticides, herbicides or other variations in cultivation procedures.

**Table 8:** Analysis of variance (ANOVA) between and within fruit samples at 95 % confidence level

Metal	Comparison	df	F_calculated_	F_critical_	Remark
Na	Between samples	4	319.66	3.48	Significant difference between sample means
	Within samples	10			
K	Between samples	4	879.70	3.48	Significant difference between sample means
	Within samples	10			
Ca	Between samples	4	1375.34	3.48	Significant difference between sample means
	Within samples	10			
Mg	Between samples	4	2539.91	3.48	Significant difference between sample means
	Within samples	10			
Fe	Between samples	4	109.50	3.48	Significant difference between sample means
	Within samples	10			
Cu	Between samples	4	98.13	3.48	Significant difference between sample means
	Within samples	10			
Zn	Between samples	4	23.80	3.48	Significant difference between sample means
	Within samples	10			
Mn	Between samples	4	242.87	3.48	Significant difference between sample means
	Within samples	10			

*df* degree of freedom of between sample and within sample

### Pearson correlation of metals between soil and fruit and between water and fruit samples

In this study, to correlate the effect of one metal concentration on the concentration of the other metal in the same sample and to correlate the effect of the same metal concentration of soil to that of fruit and water to that of fruit, the Pearson correlation matrices using correlation coefficient (r) for the samples were used. The results are given in Tables 9 and 10.

**Table 9:** Correlation matrices for metals in fruit samples (n = 5)

	Na	K	Ca	Mg	Fe	Cu	Zn	Mn
Na	1.000							
K	−0.711	1.000						
Ca	0.872	−0.719	1.000					
Mg	−0.740	0.949	−0.592	1.000				
Fe	0.028	0.645	−0.142	0.545	1.000			
Cu	0.346	0.014	−0.122	−0.288	0.31	1.000		
Zn	−0.437	0.652	−0.639	0.46	0.273	0.564	1.000	
Mn	0.622	0.052	0.307	−0.114	0.761	0.648	0.072	1.000

**Table 10:** Pearson correlation coefficient for metals in soil to fruit and water to fruit samples

Metal	Na	K	Ca	Mg	Fe	Cu	Zn	Mn
r_soil-fruit_	−0.963	−0.547	−0.676	0.574	0.499	−0.759	0.0058	0.923
r_water-to-fruit_	−0.248	−0.754	−0.275	0.265	−0.593	0.532	−0.968	0.704

r_soil-fruit_ and r_water-fruit_ are the Pearson correlation coefficients between metal level in soil and fruit and between metal level in water and fruit, respectively

There was high positive correlation for Na with (Ca and Mn), K with (Mg, Fe, Zn), Fe with Mn, and Cu with Mn and moderate correlation for Na with Cu, Ca with Mn, Mg with (Fe and Zn), Fe with (Cu and Zn) and Cu with Zn; which may arise from common anthropogenic or natural sources as well as from similarity in chemical properties. The high negative correlation for Na with (K and Mg), K with Ca, Ca with (Mg and Zn) indicate the large absorption of Na may affect the absorption of K and Mg, K may affect Ca, and Ca may affect Mg and Zn in the fruits. The other metals have weak negative or positive correlation indicating that the presence or absence of one metal affects the other to a lesser extent.

Pearson correlation coefficients for metals in soil to fruit and water to fruit samples (Table 10) indicate that Na, K and Ca have negative correlations which indicate that higher concentration of these three metals in the soil and water result in lower concentration of these metals in the fruits. Mg and Mn positively correlate to each other indicating a proportional translocation of these metals from soils and water into fruits (Al-Lahham et al., 2007). Fe correlated positively with soil and fruit but negatively with water and fruit while Cu has negative correlation between soil and fruit and positive correlation between water and fruit. Zn has negligible positive correlation between soil and fruit and strong negative correlations between water and fruit. These findings verify the dependence of metal concentrations in the fruit plant on their concentrations in soil and water media.

## Conclusions

In this study, fruits including banana, grape, guava, mandarin, and orange were analyzed for their content of Na, K, Ca, Mg, Fe, Cu, Zn, Mn, Pb, and Cd. It was found that banana (Cavendish variety) contains the highest amounts of Mg and K compared to other fruits. Therefore, banana can be a good source of Mg and K. The analysis showed that guava is rich in total ascorbic acid content. Mandarin and orange also contain appreciable amount of total ascorbic acid. The results generally indicate that the consumption of these fruits can satisfy daily intake of vitamin C of the society to some extent. The detection of cadmium in some fruits indicates its translocation from the soils that already contained concentrations above the maximum allowable limits. Water could be also the source of cadmium that contributes to the accumulation of this metal in soil and fruits.
